# Audiological Methods for Early Detection of Hearing Loss in Healthcare Worker

**DOI:** 10.3390/healthcare13101113

**Published:** 2025-05-10

**Authors:** Ramida Dindamrongkul, Thitiworn Choosong, Wandee Khaimook

**Affiliations:** 1 Department of Otolaryngology Head and Neck Surgery, Faculty of Medicine, Prince of Songkla University, Songkhla 90110, Thailand; ramida.d@psu.ac.th; 2 Department of Family and Preventive Medicine, Faculty of Medicine, Prince of Songkla University, Songkhla 90110, Thailand; cthitiwo@medicine.psu.ac.th

**Keywords:** occupational hearing loss, healthcare workers, extended high-frequency audiometry, distortion product otoacoustic emission

## Abstract

**Background**: Occupational hearing loss (OHL) is a primary concern in industrial settings. In hospitals, the healthcare workers are also exposed to noise and chemical agents, the reported hearing loss in this occupation is underestimated. Hearing examination is routinely evaluated in the range of conventional frequencies, which may not detect hearing problems early. Therefore, this study aimed to reveal the hearing thresholds among medical personnel exposed to loud noise and/or chemical environments, estimating the prevalence of hearing loss using four different audiological methods. **Methods**: One hundred and thirty-one medical personnel were recruited from different units at the same hospital and grouped into noise, chemical, and mixed exposure categories. The hearing thresholds were assessed using four audiological methods, conventional audiometry (CA), extended high-frequency audiometry (EHFA), standard frequency distortion product otoacoustic emission (DPOAE), and ultra-high-frequency DPOAE. Statistical analyses were performed using R. **Results**: Ultra-high-frequency DPOAE and EHFA showed a higher prevalence of hearing loss than CA and DPOAE. Even CA usually demonstrated hearing threshold within normal limits, this study found a notch audiogram pattern indicating a decline in hearing loss over time at frequencies of 2, 3, and 4 kHz in each age group and a sign at a frequency of 6 kHz. **Conclusions**: Evidence of hearing loss can be identified with ultra-high-frequency DPOAE and EHFA, despite conventional audiometry indicating normal hearing thresholds.

## 1. Introduction

Noise pollution negatively affects industrial workers; however, it also affects healthcare workers [1,2,3]. The World Health Organization (WHO) advises that individuals should not be exposed to noise levels exceeding 85 dB(A) within 1 h and 70 dB(A) within 24 h to reduce the adverse health effects of noise exposure. As a reference, normal conversational speech is usually equivalent to 60 dB(A); whereas, exposure to 85 dB(A) is comparable to the noise level of heavy city traffic or a loud restaurant. For hospital environments, noise levels during the daytime and nighttime should be controlled at <35 dB(A) and 30 dB(A), respectively [4]. Previous studies showed that the average of equivalent sound pressure levels in hospital ranges from 38.7 to 68.8 dB(A) during the day and 37 to 88.6 dB(A) at night, which does not comply with WHO recommendations [3]. Notably, the noise level in hospitals, including operating rooms and intensive care units, usually exceeds the limit, especially in the orthopedic department [1,2,5,6,7]. Sources of noise pollution include therapeutic instruments, bedside dialysis machines, air ventilators, printers, crying infants, public noise, and staff activities.

Occupational hearing loss (OHL) was not only associated with noise pollution but was also affected by chemical agents [8,9,10]. Individuals can inhale or absorb hazardous chemicals during work. Following this exposure, chemical agents can be absorbed into the bloodstream and various locations involving the auditory pathways.

Furthermore, medical personnel may encounter medications that pose a hearing loss risk during work. Ototoxic drugs, such as aminoglycoside antibiotics, salicylates, and platinum-based chemotherapy drugs, can cause hearing loss [11,12]. Previous studies have indicated that the effects of chemical exposure progressively increase over time in hearing disorders [13,14]. Over half of the nurses and pharmacists working in chemotherapeutic units complained of dizziness, and 75% were identified with a notch audiogram configuration at 4 and 6 kHz [10]. Following the high prevalence of cancer, medical personnel have also increased their exposure rate to ototoxic drugs, which may increase the risk of occupational diseases. Furthermore, they are frequently exposed to various factors, such as loud noise and substances that are psychologically and physiologically harmful. The long-term effects of combining these co-exposures increase the severity of emerging risks [15].

OHL is typically diagnosed using conventional audiometry (CA), which examines hearing thresholds across a full range of individuals frequencies from 0.25 to 8 kHz. A characteristic of OHL, particularly from exposure to high-frequency noise sources, is that it initially and primarily affects hearing thresholds at higher frequencies and progressively extends to lower frequencies as the hearing loss worsens [16]. CA typically assesses frequencies up to 8 kHz; therefore, it may fail to detect the early stages of extended high-frequency hearing loss. To identify cochlear damage at an earlier stage, extended high-frequency audiometry (EHFA) may require observation to detect hearing damage early at a frequency above 8 kHz. Although, EHFA is not currently used in routine clinical practice, studies have reported that testing at a frequency of 16 kHz in EHFA is highly sensitive for the early detection of noise-induced hearing loss [17,18]. However, CA and EHFA are subjective hearing tests that require individuals to respond to stimuli. Distortion product otoacoustic emission (DPOAE) and ultra-high-frequency DPOAE are useful objective tools for identifying preclinical hearing loss before it becomes detectable with CA [16,19]. According to the typical pattern of hearing loss, damage generally first occurs at high frequencies. DPOAE can assess cochlear function at frequencies up to 16 kHz with ultra-high-frequency DPOAE, providing an opportunity to detect cochlear dysfunction earlier, even before significant hearing threshold shifts are observed on CA. The DPOAE method is sensitive to detecting both early permanent hearing loss and temporary threshold shifts (TTS) in cochlea function caused by cochlear dysfunction, auditory fatigue, and ototoxic effects [19,20]. TTS is a common effect of loud noise exposure and represents a temporary reduction in hearing threshold. Although hearing may initially recover, repeated exposures can lead to progressive cochlear damage, eventually resulting in permanent hearing loss [21].

Previous studies have used DPOAE to indicate hearing changes attributable to ototoxic drugs [20,22,23]. Consequently, the earliest signs of underlying cochlear damage in medical personnel exposed to loud noise and/or chemicals, although their hearing is within normal limits when measured using the CA method, should be evaluated using EHFA and DPOAE. Therefore, we aim to explore the prevalence of hearing loss, as assessed by the following four audiological methods: CA, EHFA, standard frequency DPOAE, and ultra-high-frequency DPOAE, among medical personnel exposed to loud and/or chemical environments.

## 2. Methods

### 2.1. Participants

Medical personnel aged 21–60 years were recruited from many departments, such as medical respiratory care, emergency, dental, pharmacy, chemotherapy, anatomical pathology units, and operating rooms at Songklanagarind Hospital between March and November 2023. All participants were symmetrical hearing thresholds. Audiograms with interaural differences greater than 10 dB HL were excluded, as a standard clinical indicator of possible medical etiology. This criterion ensured the study focused specifically on the occupational impact on hearing loss. The participants were asked to identify their units, and it was grouped into three categories, loud noise, chemical, and mixed exposure, which were based on their self-rated exposure and job roles.

### 2.2. Hearing Evaluations

This cross-sectional study evaluated the hearing thresholds using four audiological methods, CA, EHFA, standard frequency DPOAE, and ultra-high-frequency DPOAE. All measurements took place in a soundproofed room. For the CA method, pulsed tones were stimulated via air conduction ranging from 0.25 to 8 kHz; whereas, EHFA was measured at 9, 10, 11.2, 12.5, 14, and 16 kHz. Normal hearing was defined as a hearing threshold ≤25 dB HL at each frequency tested. DPOAE was evoked by two pure-tone frequencies (f1 and f2) that maintained an f2/f1 ratio at 1.2, a setting to maximize the distortion product response [24]. The frequencies of f2 ranged from 552 to 7012 Hz and 8838 to 17,671 Hz for standard frequency and ultra-high-frequency DPOAE, respectively. The intensities of f1 and f2 were 65 dB SPL and 55 dB SPL, respectively. A healthy ear was considered as one having a DPOAE amplitude over −10 dB SPL at each frequency [25].

### 2.3. Statistical Analysis

Statistical analyses were performed using R software software version 4.1.2 [26] with packages “epicalc” [27], “ggplot2” [28], and “Rmisc” [29]; the unit of analysis was one person. Participant information included demographic data, such as exposure group, age group, working unit, duration of working and working hours, and the prevalence of abnormal hearing were recorded and presented in percentages. Hearing thresholds and DPOAE amplitude were presented descriptively as means ± standard deviations. To compare the prevalence of abnormal hearing and hearing thresholds among the loud noise, chemical, and mixed exposure groups, analysis of variance (ANOVA) was used. Statistical significance was set at *p*-value < 0.05.

## 3. Results

Demographic data and work-related information were collected from 131 participants using a structured self-administered questionnaire. Participants were asked to self-report their information before conducting hearing evaluations. Those participants were classified as an exposure group by self-reporting. The distribution of loud noise exposure was similar to that of mixed exposure, accounting for 51 participants; whereas, chemical exposure accounted for only 31 participants. Notably, most service sector workers in this study were employed in the operating room compared with workers in all other units. However, the highest prevalence of bilateral hearing loss was observed in the chemotherapy unit, accounting for 25%, with a trend toward greater occurrence among those who had worked for 21–30 years (Table 1). OHL develops slowly after several years of chemical exposure. The longer the working years, the greater the hearing loss observed. 

Regarding the CA method, the mean hearing threshold at each frequency was determined to be within normal limits except at 3 kHz in the right ear for those who had been working for over 31 years (Supplementary Figure S1). Conversely, the EHFA method confirmed the early detection of hearing loss in the first 10-year working group at a frequency of 14 kHz. However, the 20-, 30-, and 40-year groups were detected early at 12.5 kHz, 11.2 kHz, and 9 kHz frequencies, respectively (Supplementary Figure S2).

Age is another factor that influences OHL. Supplementary Figure S3 illustrates the accelerated hearing thresholds at 6 and 8 kHz frequencies in those aged > 40 years. Additionally, noise notches in the CA method demonstrated a similar pattern of declined progression in hearing loss over time at frequencies of 2 kHz, 3 kHz, and 4 kHz in each age group. The CA method showed the characteristics of hearing loss in occupational and older participants; however, their hearing thresholds at each frequency were mostly within normal limits. In contrast, early signs of hearing loss can be detected in those aged <40 years at a frequency of 14 kHz, and it was mostly determined at 11.2 and 10 kHz in those aged 41–50 and 51–60 years, respectively (Supplementary Figure S4).

The prevalence of hearing loss in the three exposure groups was assessed using four methods. The EHFA and ultra-high-frequency DPOAE methods identified a higher prevalence of hearing loss than CA and standard frequency DPOAE, performed at frequencies below 8 kHz (Table 2). The prevalence of hearing loss in each exposure group did not show statistically significant differences; however, each hearing examination method was individualized to detect abnormalities within the different exposure groups. The percentage of patients with abnormal hearing detected using CA, EHFA, and standard frequency DPOAE was high in the mixed, chemical, and loud exposure groups, respectively. In addition, ultra-high-frequency DPOAE methods determined abnormal hearing in each exposure group at a high prevalence rate exceeding 82%.

Using the CA method, the mean hearing threshold in each exposure group showed statistically significant differences at 0.25 kHz, 6 kHz, and 8 kHz (Table 3). This indicates that the loud exposure group had the lowest hearing threshold compared with the other groups at these frequencies. Furthermore, the pattern of noise notches in the conventional audiogram was observed at 2 kHz, 3 kHz, and 4 kHz in the loud and mixed exposure groups. However, the chemical exposure group had hearing thresholds in a flat pattern at the frequency ranging from 0.25 to 4 kHz (Supplementary Figure S5).

CA is a standard method in routine clinical practice. However, in this study, the EHFA is recognized as a useful method for the early detection of OHL due to loud noise and/or chemical exposure. Hearing thresholds at extended high frequencies changed earlier than those at conventional frequencies. The mean hearing thresholds at frequencies of 12.5 kHz, proven completely over 25 dB HL, determined abnormal hearing (Supplementary Figure S6). The hearing thresholds of those exposed to chemical agents showed more severe hearing loss than others, especially at a frequency of 9 kHz, significantly different between the loud- and mixed-exposure groups (Table 4).

Notably, under 50% of participants indicated abnormal hearing using standard frequency DPOAE, which is performed at a frequency ranging from 552 to 7012 Hz; whereas, ultra-high-frequency DPOAE recordings from 8838 to 17,671 Hz determined the major prevalence of hearing loss at approximately 88%. The mean DPOAE amplitude was found lower than −10 dB SPL at frequencies of 8970 and 11,304 Hz, which is an ultra-high-frequency DPOAE; whereas, the lower frequency showed a DPOAE amplitude of over −10 dB SPL, indicating a healthy ear (Supplementary Figure S7). Notably, the mixed-exposure group had better DPOAE amplitudes at frequencies ranging from 703 to 2827 Hz than the other exposure groups (Table 5). Additionally, the ultra-high-frequency DPOAE amplitude at each frequency was not significantly different between the exposure groups (Table 6).

This study considered that EHFA and ultra-high-frequency DPOAE were methods for the early detection of hearing loss, even when individuals had normal hearing according to the CA method. One hundred and fifteen participants with normal hearing were assessed for hearing thresholds at extended high-frequency and DPOAE amplitudes. At a frequency of 14 kHz, the hearing threshold dropped to over 25 dB HL in the three exposure groups; whereas, a frequency of 12.5 kHz showed early detection only in the chemical and loud noise exposure groups (Supplementary Figure S8). The DPOAE amplitudes slightly decreased at the standard frequency and rapidly declined at the ultra-high frequency (Supplementary Figure S9). Therefore, the significant audiological methods for the early diagnosis of OHL would be beneficial for monitoring the workplace risks and preserve hearing ability at conventional frequencies before the severity of the loss increases and affects speech communication.

## 4. Discussion

Notably, in this study, we observed that most participants had normal hearing thresholds when assessed using the CA method; however, EHFA and ultra-high-frequency DPOAE effectively detected a high prevalence of hearing loss in the early stages. CA cannot access hearing function at an extended high-frequency stimulus; however, it is widely used in routine hearing assessment, because it is readily available. Therefore, in this study, we propose the advantages of a useful audiogram configuration for CA. First, we observed a definite decline in hearing thresholds among participants aged over 40 years, particularly at frequencies of 6 and 8 kHz, compared with younger age groups. However, the hearing thresholds at 6 and 8 kHz remained better than those at other frequencies, which may have been elevated owing to occupational factors. It should be noted that age-related hearing loss can also begin after the age of 40 years, particularly at higher frequencies [30,31]. Therefore, this study is limited in that it cannot fully distinguish the contributions of occupational exposure from age-related hearing loss. However, individuals over 40 years old should be closely monitored hearing loss.

Additionally, a noise notch audiogram demonstrated a similar pattern of declined progression in hearing loss over time at frequencies of 2 kHz, 3 kHz, and 4 kHz in each age group. Previous studies have shown that a sign of noise-induced hearing loss is demonstrated by a notch audiogram at 3 kHz, 4 kHz, or 6 kHz [32,33,34]. However, this study revealed that the progression of hearing loss was exacerbated at various frequencies beyond the initial impact of noise exposure. This discovery established a biomarker for the early signs of hearing loss at a frequency of 6 kHz, which resulted in a potentially resulting from multifactorial process involving aging and noise exposure. Aging exacerbates hearing loss through the degeneration of auditory structures [35]. Although our study was cross-sectional and does not demonstrate longitudinal progression, the findings suggest that the combination of aging and occupational exposures could compound the greater severity of hearing loss, particularly among medical personnel aged over 40 years, even when their hearing thresholds remain within normal limits.

The EHFA and ultra-high-frequency DPOAE methods identified a higher prevalence of hearing loss. These measurements have become more widespread in detecting early changes in cochlear function, especially OHL [16,36,37,38]. Furthermore, based on the physiology of hearing, hair cells at the base of the cochlea are harmed more by loud noise and/or chemical agents, which first affect the hearing threshold at higher frequencies and gradually extend to lower frequencies [39].

However, according to the gradual decline in OHL, a previous study reported a two-fold prevalence of hearing loss in those exposed to noise for greater than 10 years compared with those who worked within the first 10 years [40,41,42]. The CA method may require a longer working year duration to detect hearing loss. Therefore, hearing thresholds at extended high frequencies should be evaluated for the early determination of hearing loss. This can be attributed to the research finding that the EHFA method confirmed the early detection of hearing loss in the first 10-year working group. The hearing threshold at a frequency of 14 kHz was over 25 dB HL; whereas, that of the groups with 20, 30, and 40 years of working experience were first detected at frequencies of 12.5, 11.2, and 9 kHz, respectively. Furthermore, EHFA is useful for distinguishing OHL from loud noise and/or chemical exposure. Previous studies have found elevated hearing thresholds at 14 and 16 kHz in individuals exposed to noise [38,43,44]. In our findings, the hearing threshold at a frequency of ≥12.5 kHz is an early indicator of hearing loss, particularly in medical personnel exposed to chemical agents, who appear to have higher hearing thresholds than other groups. While this study acknowledged that these current findings do not fully establish a causal relationship between chemical exposure and hearing loss, they highlighted potential attention that individuals with chemical exposure should be closely monitored in hearing. Further research is needed to explore whether cumulative chemical exposure may contribute to hearing loss.

One approach that has been used to detect hearing loss is DPOAE. Previous studies have shown that DPOAE is more sensitive than CA in detecting noise-induced hearing loss [16,24,44]. The absence of DP amplitude is directly linked to the abnormal functioning of the outer hair cells [37]. Furthermore, in this study, we found an initial reduction in the DP amplitude at a frequency of 2827 Hz, in which the DP amplitude was lower than 0 dB SPL. The magnitude of reduction was also maximally decreased lower than −10 dB SPL at frequencies of 8970 and 11304 Hz. However, the DP amplitude decreased over a higher frequency range, and over 80% of abnormal DPOAE readings were detected at ultra-high- frequencies. The prevalence of hearing abnormalities was high, as detected using the EHFA.

Previous studies have suggested that the DP amplitude of frequency over 8 kHz should be used to indicate cochlear damage, as it is more variable than lower frequency stimuli [16,45]. Furthermore, in this study, we discovered a decline in DP amplitude at ultra-high frequencies and a reduction in hearing thresholds at extended high frequencies in participants with normal hearing. These findings encourage the use of ultra-high-frequency DPOAE and EHFA measures as battery tests in audiological measurements for monitoring OHL, even when individuals do not have changes in hearing at conventional frequencies.

This study has some limitations. Notably, it was conducted on a group of medical personnel who were exposed to low noise levels in hospital. Therefore, our findings may not apply to other occupations involving exposure to loud noise and/or chemicals. However, the outcome of this study demonstrated an additional audiological measurement that should be practically used for the early detection and monitoring of OHL, especially a pre-placement assessment program of the worker instead of CA, which is clinically used in routine practice. These outcomes also raise awareness of hearing loss, even in individuals without confirmation or possibly awareness of hearing problems.

Furthermore, hearing loss risk is associated with age, duration of working years, and recreational events. In future studies, factors associated with hearing should be controlled. Sources of background noise should be differentiated to affect hearing, and the exposure level and duration should be indicated. However, patients with abnormal hearing can be monitored to determine the possibility of hearing loss.

## 5. Conclusions

The identification of hearing loss using ultra-high-frequency DPOAE and EHFA might have occurred without a reduction in the hearing threshold being observed using CA and standard frequency DPOAE. Therefore, to facilitate early identification, ultra-high-frequency DPOAE and EHFA may be useful as part of a prevention hearing screening to detect and monitor hearing change in risk occupational groups.

Even though the extended high-frequency audiogram pattern appeared generally similar across exposure groups, elevated hearing thresholds of each exposure group were observed at different specific frequencies (e.g., 11.2 kHz in the chemical exposure group and 12.5 kHz in the noise exposure and mixed exposure groups). While these findings were not sufficient to infer causality, these may suggest that clinical assessments should consider prioritizing the evaluation of hearing thresholds at 11.2 and 12.5 kHz, depending on the type of exposure group. This approach could facilitate occupational hearing screening guidelines. Early identification at these frequencies would allow for the implementation of protective measures to preserve normal hearing thresholds at conventional frequencies, preventing the loss from extending into speech frequencies, where it would have a more significant impact on communication.

## Figures and Tables

**Table 1:** Baseline characteristics of participants and prevalence of abnormal hearing in conventional audiometry method.

Characteristic.		Total		Bilateral Abnormal Hearing	
		n	%	n	%
Exposure group	Loud exposure	51	38.9	5	9.8
	Chemical exposure	29	22.1	3	10.3
	Mixed exposure	51	38.9	8	15.7
Workplace	Pharmacy department	12	9.2	1	8.3
	Operating room	48	36.6	3	6.25
	Medical respiratory care unit	10	7.6	2	20.0
	Emergency unit	17	13.0	2	11.8
	Dental unit	14	10.7	2	14.3
	Chemotherapy unit	16	12.2	4	25.0
	Anatomical pathology unit	14	10.7	2	14.3
Age group	21–30	34	26.0	3	8.8
	31–40	55	42.0	1	1.8
	41–50	30	22.9	9	30.0
	51–60	12	9.2	3	25.0
Working year group	1–10	73	55.7	6	8.2
	11–20	35	26.7	3	8.6
	21–30	7	13.0	6	85.7
	31–40	6	4.6	1	16.7
Working hour	Within 8 h	85	64.9	12	14.1
	9–12 h	40	30.5	4	10
	Over than 12 h	6	4.6	0	0

**Table 2:** The prevalence of abnormal hearing in exposure groups comparing between four audiological methods.

Audiological Methods	Side	Total (n = 131)		Loud Exposure (n = 51)		Chemical Exposure (n = 29)		Mixed Exposure (n = 51)		*p*-Value *
		n	%	n	%	n	%	n	%	
CA	Right	16	12.2	5	9.8	3	10.3	8	15.7	0.624
	Left	16	12.2	5	9.8	3	10.3	8	15.7	0.624
EHFA	Right	107	81.7	43	84.3	25	86.2	39	76.5	0.459
	Left	109	83.2	43	84.3	27	93.1	39	76.5	0.155
Standard frequency DPOAEs	Right	51	38.9	26	51.0	9	31.0	16	31.4	0.078
	Left	56	42.7	22	43.1	9	31.0	25	49.0	0.294
Ultra-high- frequency DPOAEs	Right	116	88.5	42	82.4	27	93.1	47	92.2	0.204
	Left	119	90.8	48	94.1	26	89.7	45	88.2	0.570

CA: conventional audiometry; EHFA: extended high-frequency audiometry; DPOAEs: distortion product otoacoustic emissions; *p*-value * < 0.05.

**Table 3:** Mean hearing threshold at different frequencies of conventional audiometry in each exposure group.

Frequency (kHz)	Side	Hearing Threshold (dBHL) Mean (SD)				*p*-Value *
		Total (n = 131)	Loud Exposure (n = 51)	Chemical Exposure (n = 29)	Mixed Exposure (n = 51)	
0.25	Right	14.5 (5.1)	13.8 (4.9)	16.7 (5.0)	14 (5.1)	0.031 *
	Left	16.8 (4.4)	16.2 (4.3)	18.4 (4.0)	16.5 (4.5)	0.066
0.5	Right	15.5 (4.4)	15.6 (3.7)	15.7 (4.8)	15.4 (4.8)	0.952
	Left	16.1 (4.1)	15.5 (3.8)	17.2 (4.5)	16.0 (4.1)	0.185
1	Right	16.3 (4.7)	16.1 (5.3)	16.4 (4.2)	16.5 (4.4)	0.911
	Left	16.8 (5.2)	16.4 (5.1)	17.8 (4.5)	16.8 (5.6)	0.518
2	Right	18.5 (5.3)	18.8 (6.0)	18.4 (4.6)	18.3 (5.1)	0.893
	Left	16.9 (6.1)	16.9 (6.3)	16.9 (4.9)	16.9 (6.5)	1
3	Right	19.1 (6.3)	18.6 (6.3)	17.2 (5.8)	20.6 (6.5)	0.059
	Left	18.3 (6.0)	17.8 (6.5)	17.8 (4.5)	19.1 (6.1)	0.477
4	Right	18.2 (6.5)	17.3 (6.6)	17.1 (5.8)	19.8 (6.7)	0.081
	Left	19.3 (6.4)	18.4 (6.6)	19.0 (5.2)	20.4 (6.8)	0.292
6	Right	13.3 (7.2)	11.9 (6.1)	14.3 (6.1)	14.1 (8.5)	0.194
	Left	15.1 (8.1)	12.9 (7.1)	16.2 (7.4)	16.7 (9.0)	0.047 *
8	Right	11.9 (8.8)	10.1 (8.1)	15.5 (10.4)	11.8 (8.2)	0.029 *
	Left	12.6 (9.1)	10.3 (7.8)	16.4 (11.3)	12.8 (8.3)	0.014 *

dBHL: decibels hearing level; *p*-value * < 0.05.

**Table 4:** Mean hearing threshold at different frequencies of extended high-frequency audiometry in exposure group.

Frequency (kHz)	Side	Hearing Threshold (dBHL) Mean (SD)				*p*-Value *
		Total (n = 131)	Loud Exposure (n = 51)	Chemical Exposure (n = 29)	Mixed Exposure (n = 51)	
9	Right	10.2 (11.4)	8.0 (10.0)	16.2 (15.6)	8.9 (8.8)	0.005 *
	Left	11.5 (12.3)	9.1 (10.5)	17.1 (14.9)	10.6 (11.5)	0.015 *
10	Right	15.3 (14.4)	12.3 (12.9)	20.7 (19.2)	15.3 (12.1)	0.042 *
	Left	16.0 (14.4)	14.7 (13.6)	20.5 (16.4)	14.7 (13.6)	0.157
11.2	Right	20.6 (18.3)	18.5 (16.2)	25.7 (22.3)	19.8 (17.4)	0.223
	Left	19.8 (17.2)	17.1 (14.2)	25.7 (22.4)	19.2 (16.2)	0.093
12.5	Right	29.5 (21.7)	28.4 (19.0)	35.5 (25.4)	27.1 (21.9)	0.226
	Left	29.1 (21.0)	29.1 (20.4)	34.7 (23.6)	26.0 (19.8)	0.208
14	Right	39.2 (23.0)	38.1 (21.4)	42.4 (24.1)	38.4 (24.2)	0.697
	Left	38.5 (23.2)	37.7 (23.0)	44.5 (24.2)	35.9 (22.8)	0.271
16	Right	44.6 (19.8)	44.3 (17.5)	48.6 (18.1)	42.5 (22.6)	0.420
	Left	44.8 (18.2)	45.0 (16.0)	50.8 (19.4)	41.3 (19.0)	0.078

dBHL: decibels hearing level; *p*-value * < 0.05.

**Table 5:** DP amplitude at different frequencies of DPOAEs in exposure group.

Frequency (Hz)	Side	DP Amplitude (dBSPL) Mean (SD)				*p*-Value *
		Total (n = 131)	Loud Exposure (n = 51)	Chemical Exposure (n = 29)	Mixed Exposure (n = 51)	
356	Right	3.7 (7.3)	4.3 (7.7)	3.4 (7.9)	3.4 (6.5)	0.818
	Left	3.9 (6.6)	2.7 (7.3)	5.1 (6.5)	4.5 (5.8)	0.230
444	Right	−3.3 (7.9)	−4.7 (8.7)	−1.6 (7.3)	−3 (7.4)	0.225
	Left	4.6 (6.5)	3.9 (7.3)	5.1 (6.2)	4.9 (5.8)	0.620
557	Right	5.3 (6.7)	5.4 (6.3)	4.6 (7.8)	5.6 (6.6)	0.794
	Left	5.4 (7.0)	3.9 (8.0)	6.8 (5.6)	6.2 (6.6)	0.128
703	Right	8.0 (6.7)	7.4 (6.5)	7.0 (6.7)	9.2 (6.7)	0.262
	Left	7.8 (6.4)	7.0 (7.2)	7.8 (5.6)	8.5 (6.1)	0.481
894	Right	9.6 (6.2)	9.3 (6.4)	9.3 (6.5)	10.0 (6.0)	0.786
	Left	9.4 (6.4)	8.8 (6.9)	8.8 (6.1)	10.3 (6.1)	0.451
1118	Right	8.5 (6.0)	7.4 (6.5)	7.2 (5.8)	10.2 (5.3)	0.025 *
	Left	8.3 (6.1)	8.6 (6.2)	6.8 (7.4)	8.9 (5.1)	0.314
1416	Right	6.5 (6.7)	5.5 (7.9)	4.6 (7.0)	8.5 (4.4)	0.018 *
	Left	6.6 (6.5)	7.0 (6.7)	5.0 (6.4)	7.0 (6.4)	0.332
1777	Right	5.5 (5.7)	5.2 (5.9)	4.0 (6.9)	6.5 (4.8)	0.168
	Left	4.8 (6.4)	4.6 (6.7)	4.6 (7.0)	5.0 (6.0)	0.933
2246	Right	2.2 (5.8)	1.3 (6.0)	2.0 (6.7)	3.2 (4.9)	0.253
	Left	1.7 (6.8)	0.7 (7.4)	2.2 (7.3)	2.4 (5.8)	0.416
2827	Right	−2.5 (6.6)	−3.8 (6.1)	−2.6 (7.9)	−1.1 (6.0)	0.107
	Left	−3.5 (7.0)	−4.8 (7.8)	−1.4 (5.0)	−3.2 (6.9)	0.111
3555	Right	−3.3 (7.9)	−4.7 (8.7)	−1.6 (7.3)	−3.0 (7.4)	0.225
	Left	−4.6 (8.4)	−4.9 (8.5)	−2.7 (7.1)	−5.3 (9.0)	0.380
4482	Right	−5.2 (8.0)	−5.9 (9.4)	−4.6 (7.1)	−4.7 (6.9)	0.670
	Left	−5.9 (8.0)	−5.4 (8.6)	−5.2 (6.2)	−6.6 (8.3)	0.668
5645	Right	−3.4 (8.7)	−1.7 (8.0)	−5.7 (10.1)	−3.8 (8.4)	0.129
	Left	−4.6 (9.2)	−4.1 (10.4)	−4.4 (6.9)	−5.3 (9.2)	0.809

DPOAEs: distortion product otoacoustic emissions; dBSPL: decibels sound pressure level; *p*-value * < 0.05.

**Table 6:** DP amplitude at different frequencies of ultra-high-frequency DPOAEs in exposure group.

Frequency (Hz)	Side	DP Amplitude (dBSPL) Mean (SD)				*p*-Value *
		Total (n = 131)	Loud Exposure (n = 51)	Chemical Exposure (n = 29)	Mixed Exposure (n = 51)	
7119	Right	−4.2 (8.6)	−3.9 (7.9)	−5.6 (9.9)	−3.6 (8.6)	0.582
	Left	−5.9 (9.1)	−7.3 (9.8)	−4.1 (8.9)	−5.5 (8.5)	0.307
8970	Right	−12.4 (6.1)	−11.9 (5.6)	−12.5 (6.7)	−12.8 (6.4)	0.756
	Left	−13 (6.4)	−11.5 (5.8)	−13.4 (6.1)	−14.2 (6.9)	0.103
11,304	Right	−13.7 (9.7)	−11.4 (5.6)	−15.8 (6.6)	−15 (13.5)	0.079
	Left	−13.5 (5.4)	−14.0 (5.3)	−12.9 (4.9)	−13.4 (5.9)	0.651

DPOAEs: distortion product otoacoustic emissions; dBSPL: decibels sound pressure level; *p*-value * < 0.05.

## Data Availability

Data are available on request from authors.

## Supplementary Materials

The following supporting information can be downloaded at https://www.mdpi.com/article/10.3390/healthcare13101113/s1. Figure S1: Conventional audiometry comparing between groups of working years, including under 10 years, 11–20 years, 21–30 years, and 31–40 years; Figure S2: Extended high-frequency audiometry comparing between groups of working years, including under 10 years, 11–20 years, 21–30 years, and 31–40 years; Figure S3: Conventional audiometry comparing between groups of age, including 21–30 years, 31–40 years, 41–50 years, and 51–60 years; Figure S4: Extended high-frequency audiometry comparing between groups of age, including 21–30 years, 31–40 years, 41–50 years, and 51–60 years; Figure S5: Conventional audiometry comparing between groups of exposure, such as chemical exposure, loud exposure, and mixed exposure; Figure S6: Extended high-frequency audiometry comparing between groups of exposure, such as chemical exposure, loud exposure, and mixed exposure; Figure S7: DP-gram comparing between groups of exposure, such as chemical exposure, loud exposure, and mixed exposure; Figure S8: Extended high-frequency audiometry in normal hearing participants comparing between groups of exposure, such as chemical exposure, loud exposure, and mixed exposure; Figure S9: DP-gram in normal hearing participants comparing between groups of exposure, such as chemical exposure, loud exposure, and mixed exposure.

## Abbreviations

OHL: occupational hearing loss; CA: conventional audiometry; EHFA: extended high-frequency audiometry; DPOAE: distortion product otoacoustic emissions.
