# Supplementary material for: Audiological Methods for Early Detection of Hearing Loss in Healthcare Worker
Source: Healthcare (Basel). 2025 May 10;13(10):1113. doi: 10.3390/healthcare13101113 (PMC12111715; doi:10.3390/healthcare13101113)
Supplement: Supplementary file 1 [file healthcare-13-01113-s001.zip › healthcare-3604255-supplementary.pdf]

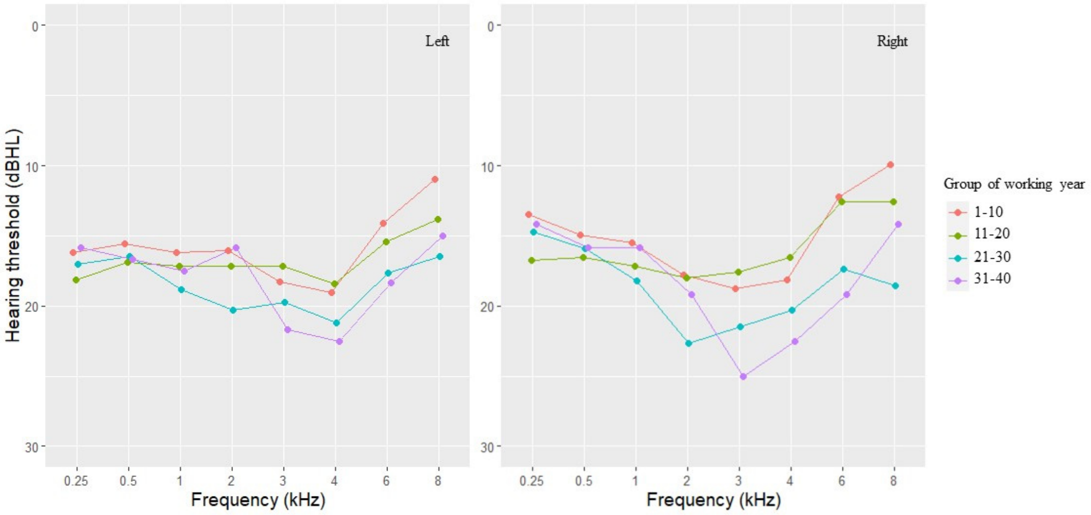

Supplementary Figure S1: Conventional audiometry comparing between groups of working years including under 10 years, 11–20 years, 21–30 years and 31–40 years

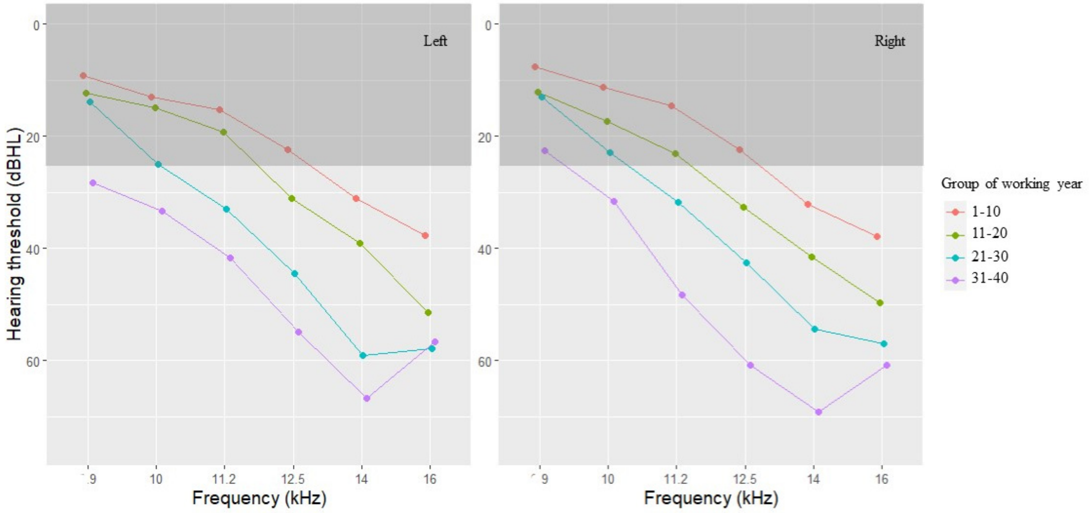

Supplementary Figure S2: Extended high-frequency audiometry comparing between groups of working years including under 10 years, 11–20 years, 21–30 years and 31–40 years

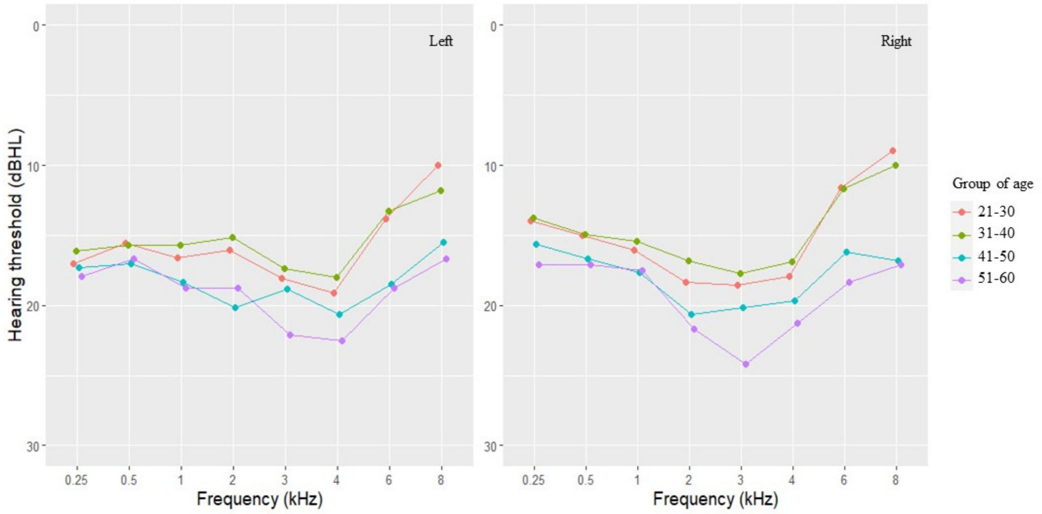

Supplementary Figure S3: Conventional audiometry comparing between groups of age including 21–30 years, 31–40 years, 41–50 years and 51–60 years

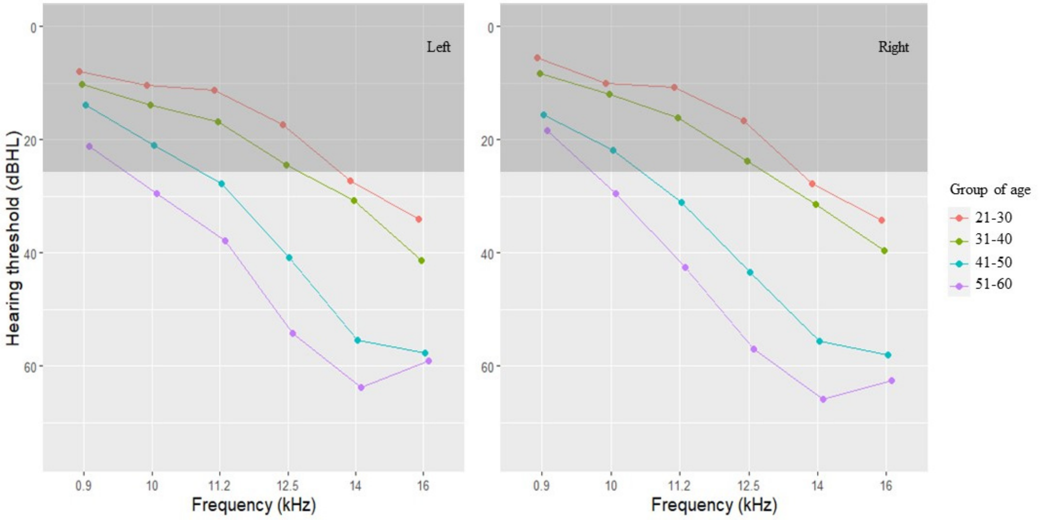

Supplementary Figure S4: Extended high-frequency audiometry comparing between groups of age including 21–30 years, 31–40 years, 41–50 years and 51–60 years

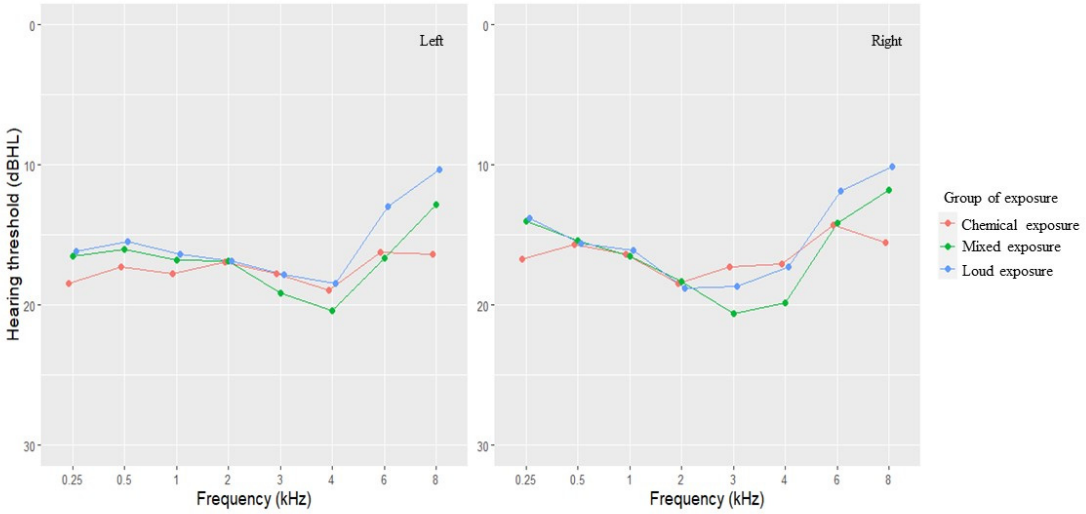

Supplementary Figure S5: Conventional audiometry comparing between groups of exposure such as chemical exposure, loud exposure and mixed exposure

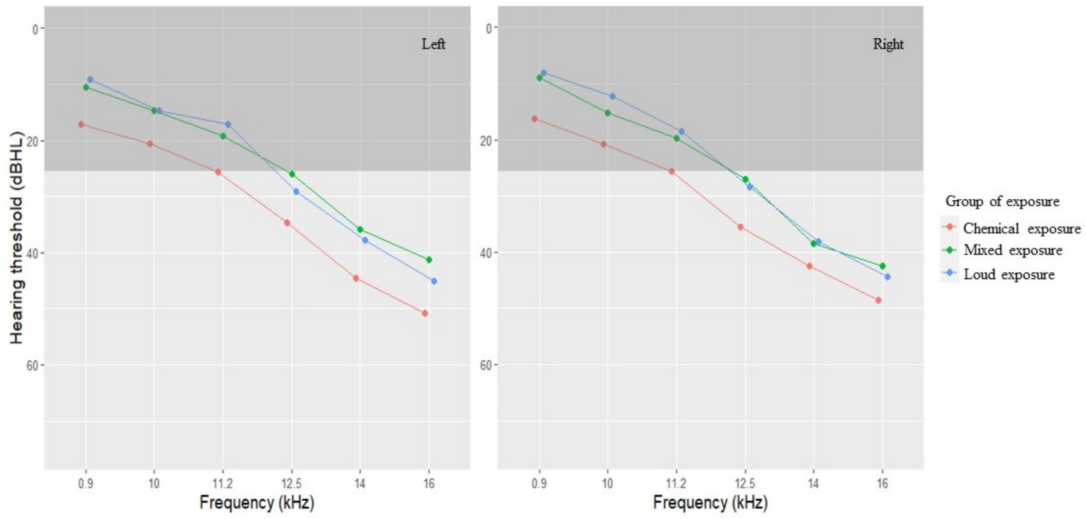

Supplementary Figure S6: Extended high-frequency audiometry comparing between groups of exposure such as chemical exposure, loud exposure and mixed exposure

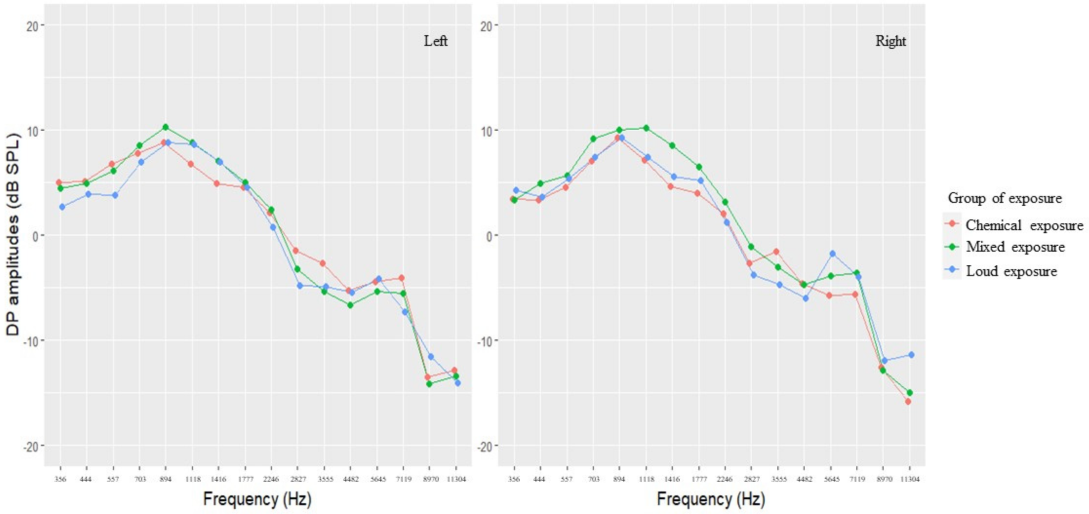

Supplementary Figure S7: DP-gram comparing between groups of exposure such as chemical exposure, loud exposure and mixed exposure

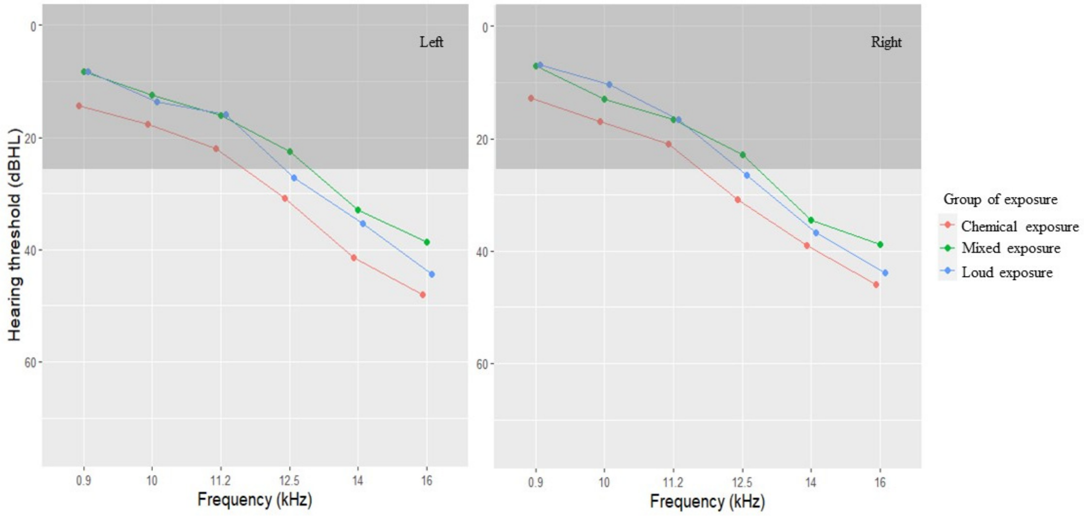

Supplementary Figure S8: Extended high-frequency audiometry in normal hearing participants comparing between groups of exposure such as chemical exposure, loud exposure and mixed exposure

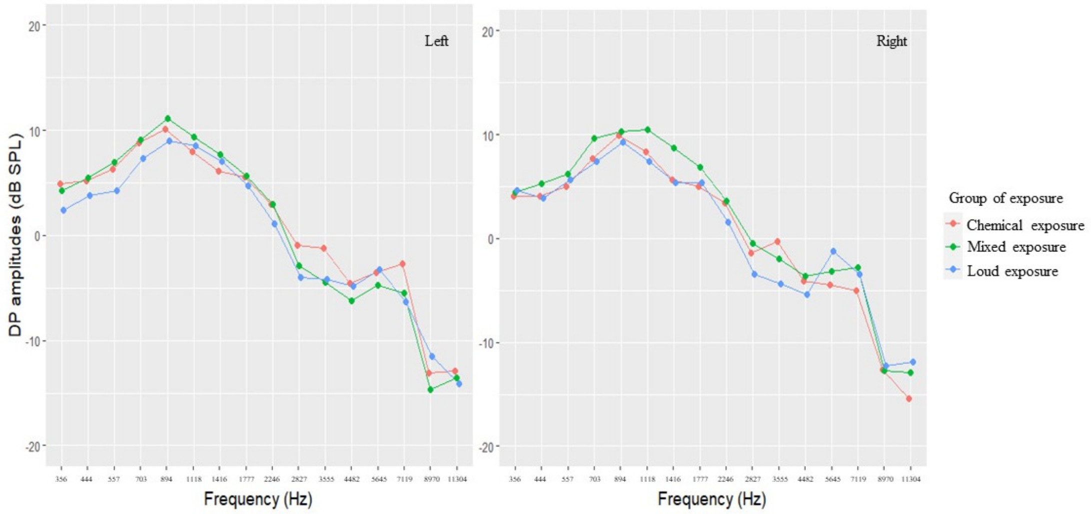

Supplementary Figure S9: DP-gram in normal hearing participants comparing between groups of exposure such as chemical exposure, loud exposure and mixed exposure
